# Adaptive visuospatial n-back training improves audiovisual integration and executive control in older adults

**DOI:** 10.3389/fpsyg.2026.1801385

**Published:** 2026-06-19

**Authors:** Yanna Ren, Binyue Gao, Heyuan Xue, Mengmeng Yang, Yulin Gao

**Affiliations:** 1 Department of Psychology, College of Humanities and Management, Guizhou University of Traditional Chinese Medicine, Guiyang, China; 2 Department of Philosophy, Jilin University, Changchun, China

**Keywords:** attention, audiovisual integration, ERP, n-back training, older adults, visuospatial working memory

## Abstract

Age-related declines in visuospatial working memory (WM), associated with the visuospatial sketchpad in Baddeley’s WM model, may impair cognitive functions including spatial orientation and visuospatial integration. Understanding these declines is important for designing interventions to support older adults’ functional independence. Although WM training has shown potential to improve WM performance and produce near transfer effects, its far transfer effects, particularly on audiovisual integration and attention, remain unclear. In this study, the training group (TG) completed five sessions of adaptive visuospatial n-back training, while the control group (CG) served as a non-contact CG. Cognitive assessments of WM, audiovisual integration, and attention were conducted before and after training for both groups. Across training sessions, task difficulty increased in the TG, suggesting learning and adaptation within the trained task. The trained 1-back task further elicited greater P200 amplitudes in the fronto-central, central, and centro-parietal regions during match trial processing in the TG, but not in the CG, indicating that adaptive visuospatial n-back training may induce task-specific learning and neural modulation. The TG also performed better than the CG on the untrained Corsi block test at posttest, suggesting a potential near transfer effect on visuospatial WM. Additionally, in the audiovisual discrimination task, the TG showed reduced audiovisual integration behaviorally after training, whereas P300-related audiovisual integration increased. In the attention network test, the TG showed greater parietal P300 amplitudes during conflict stimulus processing at posttest, despite the absence of reliable behavioral improvement. These findings suggest potential far transfer of WM training to audiovisual integration and attention, although the behavioral and electrophysiological evidence was not fully consistent across outcomes. Overall, these results suggest the plasticity of the aging brain and the feasibility of non-pharmacological cognitive interventions in mitigating age-related cognitive decline.

## 1 Introduction

Working memory (WM) is a crucially important cognitive system that allows individuals to temporarily hold and manipulate the information necessary for performing complex cognitive tasks such as learning, reasoning, and comprehension (Baddeley, 2012). It is typically conceptualized as comprising multiple components that work in concert to facilitate cognitive processing (Cowan, 2022), and the visuospatial sketchpad is a crucial component of WM, responsible for the temporary storage and manipulation of visual and spatial information (Baddeley, 2012). This subsystem plays a vital role in numerous everyday tasks, including navigation, visual search, and recognition and manipulation of objects in the environment. It also allows individuals to form mental images, understand spatial relationships, and maintain and transform visual information. Studies indicate that age-related declines in visuospatial working memory, closely associated with the functioning of the visuospatial sketchpad, can impact an individual’s ability to perform everyday tasks requiring spatial orientation and visuospatial integration; for example, older adults may find it more challenging to follow maps, arrange objects in space, or recall the visual layout of familiar environments (Old and Naveh-Benjamin, 2008). Moreover, these declines can affect complex cognitive functions, such as spatial reasoning and mental rotation, further highlighting the importance of maintaining visuospatial WM in aging populations (Goh and Park, 2009; Park and Reuter-Lorenz, 2009; Reuter-Lorenz and Park, 2014). Importantly, evidence suggests that neuroplasticity remains viable in aging, offering opportunities for targeted interventions to mitigate or even reverse such declines (Aron et al., 2022; Fraulini et al., 2024; Park and Reuter-Lorenz, 2009). For instance, Faraza et al. (2021) provided experimental evidence suggesting that behavioral training may enhance functional connectivity between key brain regions, potentially counteracting age-related decline in visuospatial WM. Their findings reported measurable training-related improvements in cognitive performance, offering support for the potential utility of targeted cognitive interventions. Complementing this empirical evidence, theoretical models such as the Scaffolding Theory of Aging and Cognition (STAC) and its revised formulation (STAC-r) suggest that cognitive aging is shaped by the dynamic interaction between age-related neural challenges, compensatory scaffolding, and life-course enrichment factors, including cognitive engagement and training experience (Goh and Park, 2009; Park and Reuter-Lorenz, 2009; Reuter-Lorenz and Park, 2014). Therefore, adaptive cognitive training may serve as a form of structured cognitive engagement that could potentially support compensatory neural recruitment and help maintain cognitive functioning in older adults.

Given the importance and neuroplasticity of visuospatial WM in everyday functioning among older adults, targeted interventions to enhance it have garnered significant interest in recent years, and many of these protocols involve targeted visuospatial WM training over the course of several days or weeks. These prior studies have primarily focused on training effects on complex cognitive functions, such as WM capacity (Dziura and Ślebarska, 2024; Richmond et al., 2011), reading and language (Richmond et al., 2011; von Bastian et al., 2013), reasoning (Dziura and Ślebarska, 2024; von Bastian et al., 2013), fluid intelligence (Blacker et al., 2017), attention (Oelhafen et al., 2013; Richmond et al., 2011; Turtola and Covey, 2021; Wang and Covey, 2020), and mathematical problem solving (Dziura and Ślebarska, 2024). However, meta-analytic evidence suggests that cognitive training, including n-back training, tends to produce more reliable gains in trained or closely related tasks, whereas transfer to untrained domains is generally smaller, less consistent, and more sensitive to methodological factors such as the nature of the control group, outcome measures, and training duration (Huang et al., 2025; Juras et al., 2024; Melby-Lervåg and Hulme, 2013; Pappa et al., 2020; Paradela et al., 2025; Sala and Gobet, 2019). Therefore, the extent to which adaptive visuospatial n-back training can transfer to low-level audiovisual integration and higher-level attentional control remains an open question that requires further investigation across multiple levels.

In addition to its structural subcomponents (e.g., phonological loop, visuospatial sketchpad, and central executive) as proposed in Baddeley’s model, WM is also a dynamic cognitive process intricately linked to low-level audiovisual integration and high-level attention networks. These systems play a crucial role in enhancing WM efficiency by filtering and organizing sensory inputs, thereby enabling the encoding, storage, and manipulation of information with greater precision (Awh et al., 2006; Chen et al., 2021; Curtis et al., 2019). Audiovisual integration is particularly important for integrating visual and auditory stimuli, forming coherent representations essential for WM function (Bigelow and Poremba, 2016), while attention networks ensure the allocation of cognitive resources needed to maintain and operate on these representations (Oberauer, 2019). Moreover, WM shares common neural and functional substrates with both audiovisual integration and attentional systems, with audiovisual integration strengthening cross-modal sensory integration processes (Covey et al., 2019; Liu et al., 2023) and attention networks enhancing WM through improved selective attention and conflict resolution (Oberauer, 2019). Emerging evidence suggests that multisensory perceptual training (Berry et al., 2010) and attention training (Peng and Miller, 2016) enhance WM performance. However, the specific impact of visuospatial WM training on audiovisual integration and attention networks remains insufficiently explored. Considering the critical role visuospatial WM plays in organizing spatial and sensory information (Awh et al., 2006), further research in this area could provide deeper insights into the mechanisms driving cognitive flexibility and far transfer effects across diverse domains.

Recent studies have predominantly employed verbal and complex visuospatial WM tasks in training protocols, involving stimuli such as numbers (Heinzel et al., 2014; von Bastian et al., 2013), letters (Dahlin et al., 2008a; Dahlin et al., 2008b; Heinzel et al., 2016; Iordan et al., 2020; Richmond et al., 2011; Turtola and Covey, 2021), words (Spironelli and Borella, 2021), and objects (Borella et al., 2010; Pergher et al., 2018). However, given the limitations of complex training paradigms, particularly for older adults, researchers have increasingly explored the potential of simpler visuospatial tasks, such as the 3 × 3 matrix paradigm. This task presents stimuli sequentially within a 3 × 3 visual grid, with each stimulus occupying one of nine spatial locations (Jaeggi et al., 2008). Verbal and complex visuospatial WM training typically combines multiple information types, including shapes, colors, and spatial positions, with verbal elements such as letters, numbers, and words. This integration imposes a high cognitive load, which may prove challenging for individuals with lower education levels or mild cognitive impairment (Kessels et al., 2010; Lövdén et al., 2020). In contrast, simpler visuospatial WM tasks like the 3 × 3 matrix are considered low-load, as they require basic spatial encoding with minimal symbolic or semantic processing. Prior research has shown that such tasks elicit lower cognitive demands and faster response times (Lavie, 2005; Luck and Vogel, 1997), making them more accessible and manageable for a broader range of participants.

To disentangle the influence of multiple cognitive components, simpler training paradigms are better suited for studying the effects of WM training on low-level audiovisual integration and high-level attention networks. For instance, Borella et al. trained older adults using a simple visuospatial WM task based on a 4 × 4 matrix (i.e., a 16-location spatial grid) and reported behavioral improvements in verbal WM, visuospatial short-term memory, inhibition, processing speed, and reasoning (Borella et al., 2014). Although attention was involved, it was assessed using a Stroop color task administered on paper and timed with a stopwatch adapted from Trenerry et al. (1989). In addition, a visuospatial WM task based on a 3 × 3 matrix was used to intervene in the cognitive function of younger adults, and near transfer benefits were exclusively observed for an untrained spatial n-back task (Minear et al., 2016). In both Borella et al. and Minear et al., only behavioral assessments were conducted, making it difficult to investigate the underlying neural mechanisms. Using EEG, Oelhafen et al. (2013) reported training-related changes in attention following dual n-back training in younger adults, as reflected by increased P300 amplitudes. More recently, Guo et al. demonstrated a transfer effect of dual n-back training on audiovisual integration, revealing increased neural activity in the frontal and central regions during early audiovisual processing (80–120 ms) post-training (Guo et al., 2023). However, the task they employed involved dual audiovisual WM using visual animal figures and corresponding auditory sounds, engaging complex cross-modal semantic processing. Due to the high cognitive demands, older adults were unable to complete the task, and only training effects for younger adults were reported. Further research is needed to determine whether, and how, visuospatial WM training can elicit transfer effects on audiovisual integration and attention in older adults. Such investigations could offer deeper insight into the mechanisms underlying age-related cognitive decline and help identify effective, targeted interventions to enhance cognitive resilience in aging populations.

Additionally, Borella et al. (2014) demonstrated that simple visuospatial tasks can effectively enhance a range of cognitive abilities. Therefore, the use of such tasks in cognitive research is valuable for gaining precise insights into fundamental cognitive processes and their enhancement through training. First, simple tasks allow for the investigation of basic sensory integration and attention networks by minimizing interference from complex information processing. This simplification not only facilitates the isolation of specific mechanisms but also makes tasks more accessible to populations with limited cognitive resources, such as older adults or individuals with cognitive impairments. Second, simple tasks offer a stable and standardized structure, improving the repeatability and control of experimental procedures, thereby enhancing the reliability and validity of the results. They also enable researchers to more accurately isolate the effects of training on specific cognitive functions without the confounding influence of more complex processes. Finally, simple tasks are easier for participants to understand and complete, increasing acceptance and engagement, which in turn supports the promotion and broader application of training programs. However, while simplicity enhances accessibility, maintaining engagement and ensuring effective transfer of training outcomes require adaptive elements that adjust task difficulty level to match individual performance levels. To this end, implementing adaptive visuospatial training programs is essential (Zini et al., 2022). Adaptive training dynamically modulates task difficulty level based on a participant’s performance, providing an optimal challenge that sustains motivation and engagement (Fraulini et al., 2024). This personalized approach not only maintains interest and reduces frustration, but also maximizes the potential for cognitive improvement by continually challenging individuals at an appropriate level (Pelosi et al., 2024). Moreover, adaptive training may facilitate the transfer of cognitive gains to real-world tasks by tailoring exercises to individual needs and abilities, ensuring that the training remains both relevant and effective (Fraulini et al., 2024). Our preliminary findings suggest that older adults struggle to complete the dual n-back task (Guo et al., 2023), highlighting the importance of developing age-appropriate training paradigms. Nevertheless, numerous studies have demonstrated that WM training can improve a range of cognitive functions, including intelligence (Amin et al., 2015; Borella et al., 2025; Guo et al., 2023; Pappa et al., 2020).

Therefore, to address these issues, the present study employed computer-based adaptive visuospatial n-back training using a 3 × 3 matrix task, following the paradigm used in Meredith et al.’s study (Minear et al., 2016). This approach aimed to investigate whether, and in what ways, WM training could enhance low-level audiovisual integration and high-level attention networks, as well as to explore the underlying neural mechanisms. Cognitive assessments were administered before and after training to evaluate training effects (visuospatial 1-back), near transfer (WM), and far transfer (audiovisual integration and attention). Previous studies have demonstrated a substantial overlap between WM and perceptual-motor training, particularly in the activation of the dorsolateral and ventrolateral prefrontal cortices. This overlap suggests that WM training may engage domain-general neural networks critical for both cognitive and perceptual processing (Salmi et al., 2018). Additionally, the episodic buffer, a key component of the Baddeley and Hitch model of WM, integrates information from the phonological loop, visuospatial sketchpad, and long-term memory into a unified representation (Baddeley, 2012). Visuospatial WM training may enhance visuospatial processing and memory maintenance, thereby strengthening the integrative function of the episodic buffer (Langerock et al., 2014), which in turn supports broader cognitive operations. Importantly, the episodic buffer plays a central role in audiovisual integration by providing the cognitive framework necessary for combining and storing multisensory information into coherent representations (Baddeley, 2012; Langerock et al., 2014). This framework enables efficient binding of auditory and visual inputs, thereby facilitating multisensory integration. Moreover, the episodic buffer and attentional resources appear to interact closely, with each system facilitating and constraining the functioning of the other (Baddeley, 2012; Langerock et al., 2014). Therefore, we hypothesized that audiovisual integration and attention networks would show training-related changes following adaptive visuospatial n-back training.

In the visuospatial 1-back training task, the P200 component provides valuable insight into early attentional allocation and perceptual processing (Covey et al., 2019), serving as a reliable index for assessing training effects in the present study. Based on previous findings that adaptive WM training can modulate attention and early-stage neural processing (Jaeggi et al., 2008), we expected that the training group would show increased P200 amplitude after training relative to the control group. In the attention network test, the N100 component, associated with early sensory processing and attentional orientation, was used to examine training-related effects on the alerting and orienting networks. We expected that the training group would show larger N100 amplitudes after training, reflecting increased increased sensitivity to salient stimuli (alerting) and more efficient attentional allocation to task-relevant spatial locations (orienting) (Jo et al., 2016; Neuhaus et al., 2010). Additionally, the P300 component, an established marker of attentional resource allocation and executive control, was analyzed under congruent and incongruent target conditions in the attention network test to assess changes in conflict monitoring and cognitive flexibility (Linden, 2005; Portin et al., 2000). We further expected that the training group would exhibit increased P300 amplitudes, reflecting enhanced executive control after training (Williams et al., 2016). In the audiovisual discrimination task, the P300 component was also examined as an index of the brain’s ability to integrate and process task-relevant multisensory information. Prior studies have shown that P300 amplitude in multisensory contexts is sensitive to training-induced changes in perceptual integration (Ren et al., 2024; Yang et al., 2018). Building on this evidence, we hypothesized that WM training would produce far transfer effects on audiovisual integration, as reflected by increased P300 amplitudes during audiovisual processing in the training group relative to the control group (Guo et al., 2023; Ren et al., 2024).

## 2 Methods

### 2.1 Participants

Fifteen participants in the TG (aged 56–72 years, mean ± SD: 61.4 ± 4.5 years) and 16 participants in the CG (aged 53–76 years, mean ± SD: 60.1 ± 5.6 years) completed the entire experiment (Table 1). In the present study, “older adults” were operationally defined as community-dwelling adults in the later adult age range, rather than by a strict age threshold. The TG and CG were matched by chronological age [*t*_(29)_ = 1.058, *p* = 0.299, *d* = 0.380], sex [*χ^2^* = 1.569, *p* = 0.210] and education [*χ^2^* = 4.839, *p* = 0.304]. All participants were cognitively healthy, had MMSE scores greater than 26, and were not taking any medications that might have central effects. Before the experiment, all participants provided informed consent, which was approved by the Medical Ethics Committee of the Second Affiliated Hospital of Guizhou University of Traditional Chinese Medicine. To minimize expectancy effects, both groups completed tasks matched in structure, duration, and schedule, with strict control over session timing to ensure procedural consistency.

**Table 1:** Demographic data for age (standard error of the mean), sex, and education.

Characteristics	All participants	Training group	Control group
*N*	31	15	16
Age (year)	61.1 (5.1)	61.4 (4.5)	60.1 (5.6)
Sex (female)	15	9	6
Education (level)			
Primary school	7	2	5
Middle school	10	4	6
High school	12	8	4
University	3	2	1

### 2.2 Apparatus, stimuli, and procedure

All stimulus presentation and behavioral data collection in the current study were managed using E-prime 3.0 software (Psychology Software Tools, Inc., Pittsburgh, PA, United States). All EEG data were recorded using the BrainVision actiCHamp Plus system (Brain Products GmbH, Gilching, Germany), with 32 Ag/AgCl electrodes mounted on an electrode cap (actiCAP GmbH, Herrsching, Germany). In the present study, all of the visual stimuli were presented on a computer monitor (Dell, E2213c) positioned 60 cm in front of the participants, and the auditory stimuli were presented through two speakers (Edifier, R19U) behind the monitor at approximately 60 dB SPL.

Adaptive visuospatial n-back training was conducted over five consecutive days in a sound-attenuated, electrically shielded, and dimly lit test room. On Day 1, prior to the commencement of training, a cognitive assessment (pretest) was administered to both the TG and CG. This assessment included tasks evaluating WM, attention networks, and audiovisual integration. Following the pretest, participants in the TG completed adaptive visuospatial n-back training for five consecutive days. The CG did not receive any intervention and remained non-contact throughout the study period. On Day 5, after the final training session, all participants in both groups completed the same battery of cognitive assessments as at pretest (posttest). To mitigate the potential effects of fatigue or reduced motivation in the TG, participants were provided with sufficient rest prior to the posttest. A mandatory minimum rest period of 30 min was enforced, with the option for extended rest based on individual needs; no upper time limit was imposed. All cognitive assessments were conducted in a controlled laboratory environment (Department of Psychology, Guizhou University of Traditional Chinese Medicine, China). Behavioral data were collected for the training task and the Corsi block test, whereas both behavioral and EEG data were recorded for the audiovisual discrimination task and attention network test.

#### 2.2.1 Adaptive visuospatial working memory training

**Figure 1:**
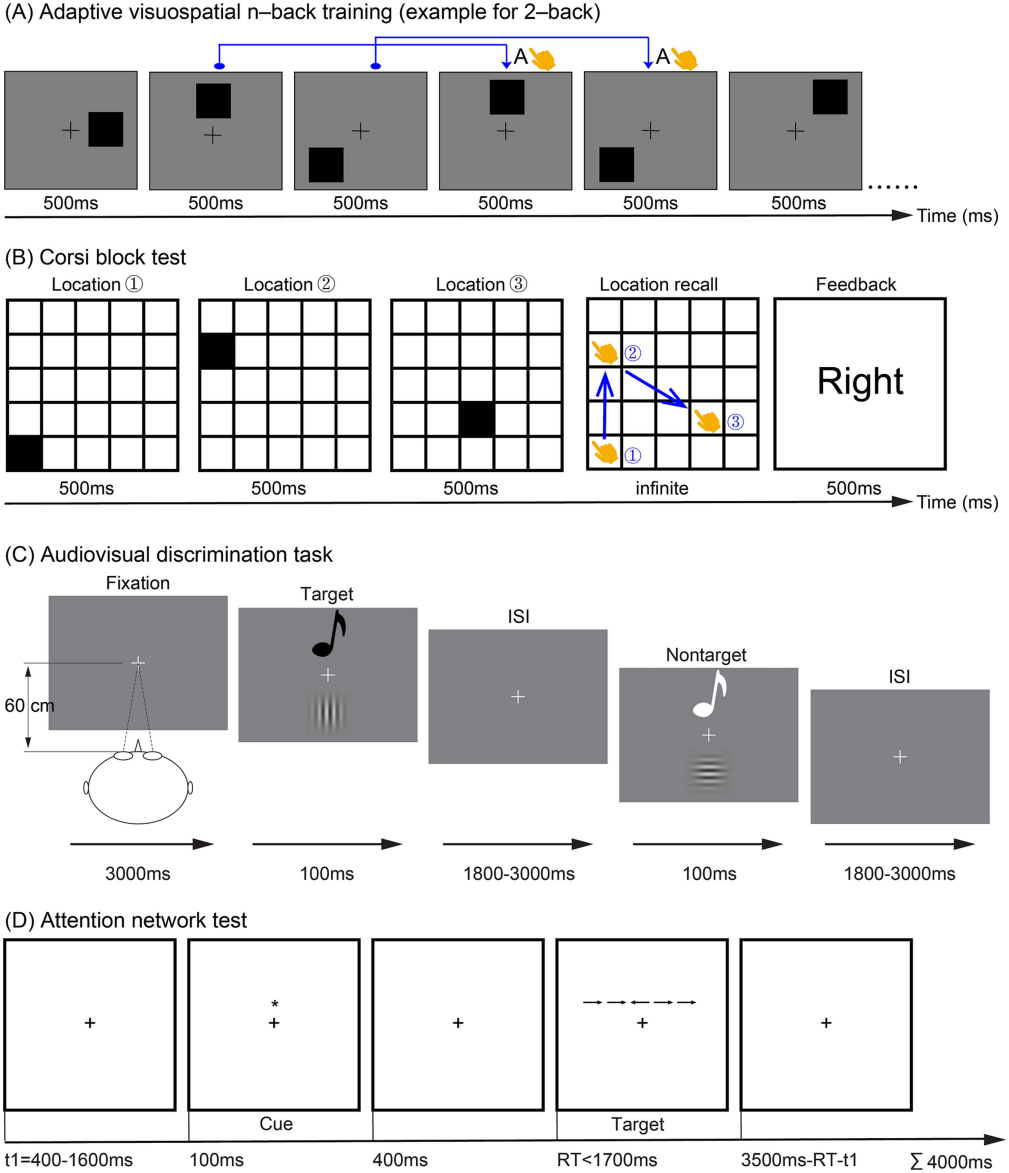
**(A)** Adaptive visuospatial n-back training scheme, illustrated with a 2-back task requiring a button press A if the current stimulus matches the one from two trials earlier. **(B)** Corsi block test, where participants replicating sequences of tapped blocks, with difficulty gradually increasing. **(C)** Example sequence of audiovisual target and nontarget stimuli in the discrimination task. The musical note is shown for illustration only and was not displayed during the experiment; participants heard the auditory tone without visual representation. **(D)** Experimental paradigm for the attention network test, featuring a spatial cue and an incongruent target condition.

The 3 × 3 matrix task adapted from a previous study (Jaeggi et al., 2008) was applied. A fixation “+” was presented at the center of a gray computer screen (RGB: 192, 192, 192), and a visuospatial black square with a 2.5° visual angle subsequently appeared randomly and consecutively in one 3 × 3 matrix surrounding the fixation (Figure 1A). Each visuospatial square was presented for 500 ms, followed by an interstimulus interval (ISI) of 2,500 ms. Participants were instructed to determine whether the current stimulus matched the stimulus presented *n* trials prior. For example, in the 2-back condition, participants were instructed to match the current stimulus with one presented two trials earlier and to press the “A” key if the stimuli matched but to withhold if they did not match (Figure 1A). Each block consisted of *n* initial trials required to establish the n-back sequence, followed by 20 scored trials, including 6 match trials and 14 non-match trials. Because responses were not required for the initial *n* trials, these trials were not included in the calculation of accuracy. The *n* value is adjusted on basis of the participant’s performance after each block: if the accuracy is ≥ 90%, the *n* value increases by 1; if the accuracy is ≤ 70%, the *n* value decreases by 1; and if the accuracy rate is between 70 and 90%, the *n* value remains unchanged. The initial training starts with a 1-back task. A training session lasts approximately 60 min and includes 40 blocks.

#### 2.2.2 Cognitive assessment

##### 2.2.2.1 Training effect

The visuospatial 1-back task was employed to evaluate the training effect. The procedure was identical to that used during training (*n* = 1) and consisted of 10 blocks, including 60 match trials and 140 nonmatch trials.

##### 2.2.2.2 Near transfer

The visuospatial WM span task was adapted from the classic Corsi spatial span paradigm. As illustrated in Figure 1B, a 5 × 5 matrix (each square subtending 2.5° of visual angle) was displayed at the center of a white computer screen. A sequence of black squares then appeared randomly within the matrix. Following the sequence presentation, participants were instructed to use the left mouse button to click on the squares in the exact order in which they had appeared. Immediate feedback was provided after each trial, indicating whether the response was correct or incorrect. The task progressed through levels involving 3 to 9 squares. Each level consisted of five trials. If a participant correctly recalled the sequence in at least one of the five trials, that level was considered achieved. The highest span level successfully completed was recorded as the participant’s visuospatial WM span.

##### 2.2.2.3 Far transfer

###### 2.2.2.3.1 Audiovisual integration

As in our previous study (Ren et al., 2023), the audiovisual discrimination task was used to evaluate audiovisual integration. The visual target stimulus was a vertical Gabor patch with 10% contrast and 1.5 cycles per degree spatial frequency, while the visual nontarget stimulus was a horizontal Gabor patch with the same contrast and spatial frequency (5° visual angle). The auditory target stimulus was a 1,000 Hz sinusoidal tone, and the auditory nontarget stimulus was a 500 Hz sinusoidal tone, each with a 10 ms rise/fall cosine gate. Audiovisual stimuli consisted of the simultaneous presentation of matched visual and auditory stimuli, and both the visual and auditory components were either targets or nontargets. No mismatched audiovisual combinations were included in the task. A white fixation (“+”) was continuously presented at the center of a gray background screen (RGB: 192, 192, 192). Visual stimuli were presented 5° below the fixation, and auditory stimuli were delivered through speakers positioned behind the screen, spatially co-located with the visual stimuli. Auditory, visual, and audiovisual stimuli were presented in a randomized order for 100 ms, with an ISI ranging from 1800 to 3,000 ms (Figure 1C). Participants were instructed to respond as quickly and accurately as possible by pressing the left mouse button whenever a target stimulus (auditory, visual, or audiovisual) was presented. Each type of nontarget stimulus was presented in 60 trials, and each target stimulus in 20 trials. In total, the experiment included 240 trials (180 nontarget and 60 target trials) divided into two blocks, with a self-paced break between blocks.

###### 2.2.2.3.2 Attention

The experimental procedure used in this study followed the protocol described by Jo et al. (2016), and the stimulus properties were based on the original paradigm developed by Fan et al. (2002). Each trial began with a black fixation (“+”) presented for a randomly varying duration between 400 and 1,600 ms. Subsequently, one of the visual cues appeared for 100 ms, signaling the imminent appearance of the target stimulus. After a 400 ms delay, the target stimulus was presented either above or below the fixation. Participants were instructed to respond to the direction of the central arrow by pressing the left key if it pointed left and the right key if it pointed right. The target stimulus remained on the screen until the participant responded or for a maximum of 1700 ms. After the stimulus disappeared, the fixation was displayed for the remaining duration to ensure that the total length of each trial was 4,000 ms (Figure 1D). The target stimulus consisted of five horizontally aligned arrows, as described by Fan et al. (2002). In the congruent condition, all five arrows pointed in the same direction; in the incongruent condition, the central arrow pointed in the opposite direction to the flankers. Participants were instructed to maintain their gaze on the fixation throughout the experiment and to respond as quickly and accurately as possible to the direction of the central arrow. Prior to the formal experiment, participants completed a practice session and were required to achieve accuracy rate of at least 80% before proceeding. The task included 48 trials for each cue type (center cue, spatial cue, and no cue) in a 1:1:1 ratio, and 72 trials for each target type (congruent and incongruent) in a 1:1 ratio. In total, there were 144 trials, divided into two blocks with a self-paced break between them.

### 2.3 Data analysis

Before conducting parametric analyses, the normality of the dependent variables was examined using the Shapiro–Wilk test. For repeated-measures ANOVAs, the sphericity assumption was assessed, and Greenhouse–Geisser corrections were applied when this assumption was violated. Post hoc pairwise comparisons were adjusted using the Bonferroni correction. Effect sizes were reported as partial eta squared (*η_p_^2^*) for ANOVA effects and Cohen’s *d* for post hoc pairwise comparisons. When the assumptions for parametric analyses were not met, nonparametric tests were used as appropriate. Specifically, the Mann–Whitney U test was used for between-group comparisons, and the Wilcoxon signed-rank test was used for within-group pre-post comparisons. Effect sizes for nonparametric tests were calculated as r = Z/√N.

#### 2.3.1 Behavioral data

Accuracy was calculated consistently across all behavioral tasks as the number of correct responses divided by the total number of experimental trials. Correct responses were defined according to the requirements of each task. Where applicable, hits and correct rejections were coded as correct responses, whereas misses, false alarms, incorrect key presses, and no-response trials were coded as incorrect responses. Response times were analyzed only for the audiovisual discrimination task and the attention network test. Response time analyses were based on correct response trials after excluding trials with response times outside ±3 SD of each participant’s mean within each task. For the WM task, performance was indexed by accuracy only, because the task emphasized response accuracy rather than response speed.

##### 2.3.1.1 Adaptive visuospatial n-back training

The task difficulty levels for each participant during training were analyzed using one-way ANOVA to evaluate the benefits of the n-back task. Post hoc pairwise comparisons were conducted to identify specific differences between difficulty levels.

##### 2.3.1.2 Cognitive assessment

###### 2.3.1.2.1 Training effect

Accuracy in the visuospatial 1-back was measured and analyzed using 2 (group: TG, CG) × 2 (test: pre, post) mixed-design ANOVA.

###### 2.3.1.2.2 Near transfer

The highest number of correctly recalled sequences in the Corsi block test was recorded and analyzed using the Mann–Whitney U test for between-group comparisons and the Wilcoxon signed-rank test for within-group comparisons.

###### 2.3.1.2.3 Far transfer—audiovisual integration

Both accuracy and response time for each participant and each target stimulus were recorded in the audiovisual discrimination task. The data were analyzed using a 2 (group: TG, CG) × 3 (stimulus: auditory, visual, audiovisual) × 2 (test: pre, post) mixed-design ANOVA. Additionally, audiovisual integration was quantified using the race model by cumulative distribution functions (CDFs), as our previous study (Ren et al., 2023). The race model (P_RM_) is a statistical prediction model [P_RM_ = (P_A_ + P_V_)-P_A_ × P_V_] based on the CDFs of the unimodal auditory condition (P_A_) and unimodal visual condition (P_V_), allowing a direct comparison with the bimodal audiovisual condition (P_AV_). P_A_, P_V_, and P_AV_ are the probability of responding within a given time in a unimodal auditory trial, unimodal visual trial, and bimodal audiovisual trial, respectively. If P_RM_ was significantly different from P_AV_, audiovisual integration is considered to occur. To calculate this, each subject’s race model CDF was subtracted from their audiovisual CDF in 10-ms time bins, producing a probability difference curve (Ren et al., 2023). Following previous studies, the peak benefit of this difference curve was used as the index of audiovisual integration. These values were analyzed using a 2 (group) × 2 (test) mixed-design ANOVA.

###### 2.3.1.2.4 Far transfer—attention

The response time for each participant under each cue and target condition were recorded in the attention network test and analyzed using a 2 (group: TG, CG) × 3 (cue: center, no, spatial) × 2 (target: congruent, incongruent) × 2 (test: pre, post) mixed-design ANOVA. The attention network indices were subsequently calculated separately (Jo et al., 2016; Neuhaus et al., 2010): alerting (RT_no cue_ – RT_center cue_), orienting (RT_center cue_ – RT_spatial cue_), and executive control (RT_incongruent cue_ – RT_congruent cue_). Each index was then submitted to a 2 (group) × 2 (test) mixed-design ANOVA.

#### 2.3.2 EEG data

##### 2.3.2.1 EEG recording

Vertical eye movements and eye blinks were measured by acquiring EOG data from an electrode placed approximately 1 cm below the participant’s left eye, and horizontal eye movements were measured by acquiring the EOG signal from one electrode placed approximately 1 cm from the outer canthi of the left eye. The reference electrode was Fz. The impedance was maintained below 5 kΩ, and the digitized sample frequency was 1,000 Hz.

##### 2.3.2.2 Preprocessing

All the EEG data was imported and processed with MATLAB R2013b (MathWorks, Inc., Natick, MA, United States) with the open source EEGLAB toolboxes (Swartz Center for Computational Neuroscience, La Jolla, CA, United States). The EEG electrodes were positioned according to the 32-channel montage of the international 10/20 system. First, the vertical and horizontal EOG were removed, and the data were re-referenced to the bilateral mastoids (TP9 and TP10). The original reference data was recovered to Fz. The remaining continuous EEG data were bandpass filtered from 0.1 to 100 Hz and notch filtered from 49 to 51 Hz. The data were segmented into epochs of 1,200 ms (200 ms pretarget and 1,000 ms posttarget). Independent component analysis (ICA) was applied to remove artifacts, and trials with EEG amplitudes exceeding ±100 μV were excluded automatically from further analysis. Baseline correction was then performed by subtracting the mean voltage of the 200 ms pretarget interval from each data point within the posttarget epoch. To enhance the target components, the data were digitally filtered with a bandpass filter ranging from 1 to 30 Hz, followed by baseline correction using the −100 to 0 ms pretarget interval. The data were then averaged for each stimulus type at the individual level. Grand-averaged waveforms were subsequently obtained across all participants for each stimulus type at each electrode.

##### 2.3.2.3 Training effect

EEG data from 60 match trials in the 1-back task were analyzed for each participant. On average, trials were excluded due to artifacts as follows: at pretest, 9.2% (TG, 48–59 trials retained) and 8.4% (CG, 52–60 trials); at posttest, 7.3% (TG, 46–60 trials) and 9.1% (CG, 45–60 trials). Based on previous studies (Covey et al., 2019; Cowan, 2022), we focused on the P200 component, which typically peaks around 140 ms. Four regions of interest (ROIs) were defined: frontal (F7, F3, Fz, F4, F8), fronto-central (FC5, FC1, FC2, FC6), central (C3, Cz, C4), and centro-parietal (CP5, CP1, CP2, CP6). Mean amplitudes were calculated in consecutive 10-ms time bins within the 100–180 ms window, following the method outlined by Keil et al. (2014). Significant group differences were observed within the 120–160 ms interval. Mean P200 amplitudes across this window were averaged across electrodes within each ROI and submitted to one-way ANOVAs. The results revealed no significant lateralization effects (all *ps* ≥ 0.174); therefore, the electrodes showing the highest activity within each ROI (Fz for frontal, FC1 for fronto-central, Cz for central, and CP1 for centro-parietal) were selected for further analysis. The mean P200 amplitude (120–160 ms) was then analyzed using a mixed-design ANOVA with group (TG, CG), ROI (Fz, FC1, Cz, CP1), and test (pre, post) as factors.

##### 2.3.2.4 Far transfer

###### 2.3.2.4.1 Audiovisual integration

Consistent with previous studies, EEG elicited by nontarget stimuli (60 trials each for auditory, visual, and audiovisual stimuli) were analyzed to avoid contamination from activity related to targets and motor responses (Ren et al., 2023; Talsma et al., 2010). On average, trials were excluded due to artifacts as follows: at pretest, 5.2% (TG, 53–60 trials retained) and 6.1% (CG, 49–60 trials); at posttest, 5.7% (TG, 51–60 trials) and 4.9% (CG, 50–60 trials). The P300 component was utilized to assess the transfer effects of WM training on audiovisual integration (Guo et al., 2023; Ren et al., 2023). P300-related audiovisual integration was calculated as the amplitude difference between the ERP elicited by bimodal audiovisual stimuli (ERP_audiovisual_) and the sum of the ERPs elicited by unimodal auditory and visual stimuli [ERP_(auditory + visual)_], that is, [ERP_audiovisual_ – ERP_(auditory + visual)_] (Ren et al., 2023; Talsma et al., 2010). In line with a previous study (Ren et al., 2023), five ROIs were defined: left anterior (F3, FC5, FC1), right anterior (F4, FC6, FC2), central (C3, Cz, C4), left posterior (P3, CP5, CP1), and right posterior (P4, CP6, CP2). The mean amplitude of all electrodes within each ROI was used for analysis. For each group, mean amplitudes were calculated across consecutive 10-ms time bins within the 300–600 ms window at each ROI, following the approach described by Keil et al. (2014). A one-way ANOVA was performed to examine group differences, and a cluster of consecutive time bins from 370–430 ms showing significant group effects was identified. The average P300 amplitudes of the difference wave [ERP_audiovisual_ – ERP_(auditory + visual)_] within each ROI across 370–430 ms used to evaluate audiovisual integration (*Δ* amplitude) by three-factor (group, test, ROI) mixed-design ANOVA. As shown in prior studies, more negative values in this difference was reflect stronger audiovisual integration, due to reduced ERP responses in the audiovisual condition relative to the auditory-only and visual-only conditions (Guo et al., 2023; Ren et al., 2023; Talsma et al., 2010).

###### 2.3.2.4.2 Attention

In parallel with the behavioral calculations, the ERP-related alerting and orienting effects were computed using 48 trials per cue type, while executive control effects were calculated using 72 trials per target type, as follows (Jo et al., 2016; Neuhaus et al., 2010): alerting amplitude = amplitude _center cue_ – amplitude _no cue_, orienting amplitude = amplitude _spatial cue_ – amplitude _center cue_, and executive control amplitude = amplitude _congruent target_ – amplitude _incongruent target_. Consistent with previous studies (Jo et al., 2016; Neuhaus et al., 2010), two ERP components were analyzed: Target N100 (100–300 ms posttarget onset), associated with alerting and orienting effects, measured at occipital (O1, Oz, O2) and parietal (P7, P3, Pz, P4, P8) regions; and target P300 (300–600 ms posttarget onset), linked to executive control processes, measured at parietal sites.

###### 2.3.2.4.3 Target N100 alerting and orienting effects

**Figure 2:**
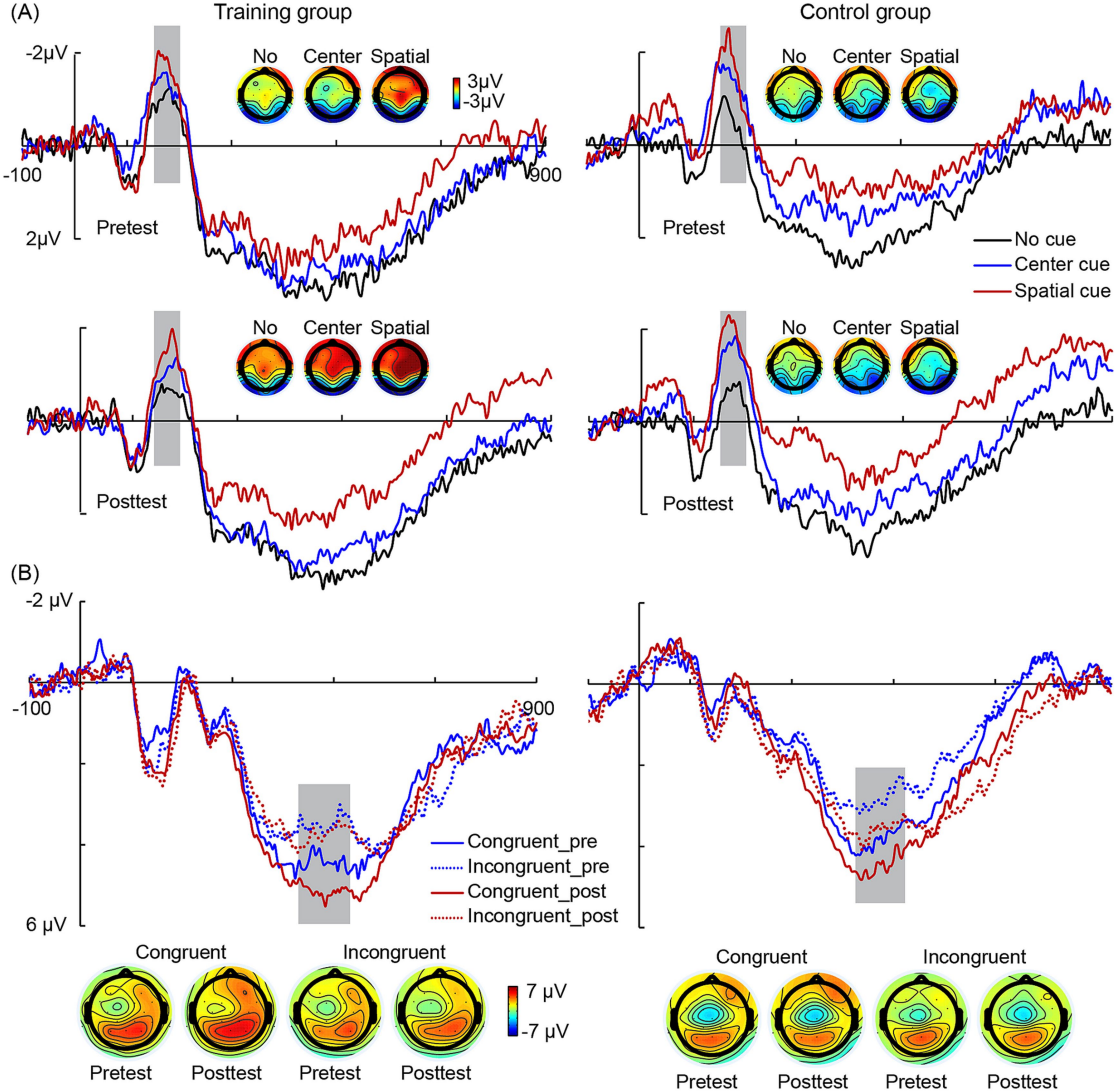
Grand average event-related potential for target-locked N100 in the right parieto-occipital electrodes (P3, P4, P7, P8, O1, O2) and its topographic distribution in the 150–200 ms time window **(A)**. The target-locked P300 amplitude across the 420–520 ms time interval at Pz was greater at posttest than at pretest for the training group **(B)**.

On average, trials were excluded due to artifacts as follows: at pretest, 7.9% (TG: 39–48 trials retained); and 6.8% (CG: 43–48 trials), and at posttest, 7.8% (TG: 39–46 trials) and 7.5% (CG: 41–47 trials). Mean amplitudes were calculated for each group across consecutive 10-ms time bins within the 100–300 ms interval at occipital and parietal regions, following Keil et al. (2014). Based on these results, the 150–200 ms time window was selected for further analysis (Figure 2A). The maximum amplitude of the N100 component was observed bilaterally in the parieto-occipital regions. Pointwise running *t*-tests with Bonferroni correction revealed no significant differences between homologous electrode pairs (P3 vs. P8, P4 vs. P7, O1 vs. O2; *all p*s ≥ 0.548). Therefore, in line with previous literature (Neuhaus et al., 2010), mean amplitudes of the target-related N100 component within the 150–200 ms window after target onset over bilateral parieto-occipital sites were used to examine attentional processing by a 2 (group) × 2 (test) × 3 (cue) mixed-design ANOVA.

###### 2.3.2.4.4 Target P300 conflict effect

On average, trials were excluded due to artifacts as follows: at pretest, 7.9% (TG: 54–71 trials retained) and 6.1% (CG: 61–72 trials); at posttest, 7.8% (TG: 53–69 trials) and 7.5% (CG: 55–70 trials). Mean amplitudes were computed for each group and each electrode across consecutive 10-ms time bins within the 300–600 ms window, using a one-way ANOVA (Keil et al., 2014). Time bins showing significant group differences within the 420–520 ms range were identified and used to quantify the conflict effect (Figure 2B). To determine the peak of the P300 component, a pointwise running *t-test* with Bonferroni correction was applied across parietal electrodes (P7, P3, Pz, P4, P8). The grand-average waveform showed that Pz exhibited the highest P300 amplitude among these parietal electrodes. Therefore, mean P300 amplitudes at Pz were used to evaluate conflict-related neural activity by a 2 (group) × 2 (test) mixed-design ANOVA.

## 3 Results

### 3.1 Behavioral results

#### 3.1.1 Adaptive visuospatial n-back training

**Figure 3:**
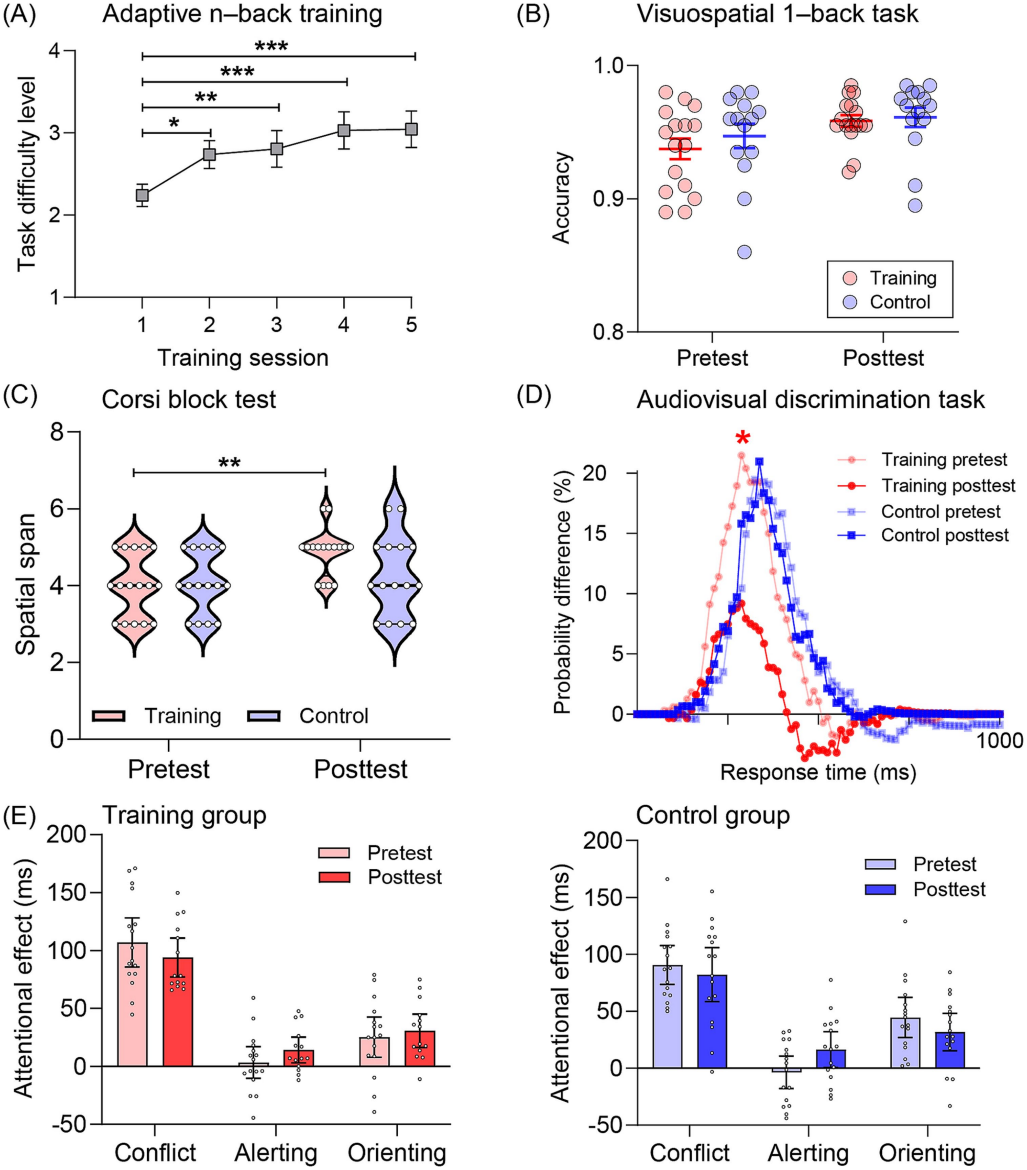
**(A)** Accuracy on the adaptive visuospatial n-back task across training sessions, **(B)** accuracy on the visuospatial 1-back task, **(C)** spatial spans on the Corsi block test, **(D)** audiovisual integration, and **(E)** attention network scores at pre and posttest for the training and control groups. The error bars represent the standard error of the mean (SEM). ^*^p < 0.05, ^**^p < 0.01, ^***^p < 0.001.

There was a significant main effect of difficulty level [*F*(4, 56) = 12.496, *p* < 0.001, *η_p_^2^* = 0.472] (Figure 3A). Post hoc analysis showed significant improvements in performance relative to Session 1 (*all ps* ≤ 0.017); however, no significant differences were found between later sessions, suggesting that the most pronounced gains occurred after the first session.

#### 3.1.2 Cognitive assessment

##### 3.1.2.1 Training effect

There was a significant main effect of test [*F*(1, 26) = 5.677, *p* = 0.025, *η_p_^2^* = 0.179], indicating higher accuracy at posttest compared to the pretest (Figure 3B). However, no significant main effect of group [*F*(1, 26) = 0.342, *p* = 0.564, *η_p_^2^* = 0.013] or interaction between group and test [*F*(1, 26) = 0.301, *p* = 0.588, *η_p_^2^* = 0.011] was observed.

##### 3.1.2.2 Near transfer

The Mann–Whitney U test for the Corsi block test revealed no significant difference between the TG and CG at pretest [*U* = 84.00, *p* = 0.164, *r* = 0.252], whereas the TG showed higher scores than the CG at posttest [*U* = 65.00, *p* = 0.031, *r* = 0.387]. The Wilcoxon Signed-Rank test for the CG found no significant difference between the pre and posttest scores [*Z* = −0.576, *p* = 0.565, *r* = 0.144], however, there was a significant increase in spatial spans at pretest compared with the posttest for the TG [*Z* = −3.276, *p* = 0.001, *r* = 0.846] (Figure 3C). These results might demonstrate an improvement in WM following adaptive visuospatial n-back training.

##### 3.1.2.3 Far transfer

###### 3.1.2.3.1 Audiovisual integration

For accuracy, significant main effects of group, stimulus, and test were observed, however, no significant interactions among these factors were found (Table 2). For response time, significant main effects of stimulus and test were observed (Table 2). In addition, a significant group × test interaction was found (Table 2). Post hoc analysis revealed that responses were faster at posttest compared to the pretest for the TG in response to visual stimulus [*t*_(14)_ = 2.165, *p* = 0.047, *d* = 0.559] and auditory stimulus [*t*_(14)_ = 2.430, *p* = 0.029, *d* = 0.627], but remained comparable for audiovisual stimulus [*t*_(14)_ = 1.241, *p* = 0.235, *d* = 0.321]. In contrast, the CG showed no significant differences in response to any stimulus (all *ps* ≥ 0.159). Additionally, at posttest, the response of TG to auditory stimulus was significantly faster compared to the CG [*t*_(29)_ = 1.942, *p* = 0.026, *d* = 0.590], while the response to visual stimulus showed a trend toward faster responses [*t*_(29)_ = 1.187, *p* = 0.061, *d* = 0.427] that did not reach statistical significance. However, no significant differences were observed for any other stimulus between the TG and CG (all *ps* ≥ 0.258). These results indicate that perceptual sensitivity was improved through training.

**Table 2:** Mixed-design ANOVA results for accuracy and response time in audiovisual discrimination task.

Main Effects and Interactions	*df*	F	*p*	*η_p_^2^*
Accuracy				
Group	1, 29	6.348	**0.017**	0.282
Test	1, 29	18.134	**<0.001**	0.385
Test × Group	1, 29	0.182	0.673	0.006
Stimulus	2, 58	15.496	**< 0.001**	0.348
Stimulus × Group	2, 58	0.648	0.512	0.022
Test × Stimulus	2, 58	0.660	0.514	0.022
Test × Stimulus × Group	2, 58	0.385	0.673	0.013
Response time				
Group	1, 29	0.702	0.409	0.024
Test	1, 29	5.089	**0.032**	0.149
Test × Group	1, 29	4.803	**0.049**	0.184
Stimulus	2, 58	110.85	**<0.001**	0.792
Stimulus × Group	2, 58	1.198	0.300	0.040
Test × Stimulus	2, 58	0.196	0.662	0.007
Test × Stimulus × Group	2, 58	1.289	0.280	0.043

Values shown in bold denote statistically significant differences (*p* < 0.05).

Analysis for the audiovisual integration (Figure 3D) revealed a significant group × test interaction [*F*(1, 29) = 4.719, *p* = 0.037, *η_p_^2^* = 0.198], with no main effects of group or test (all *ps* ≥ 0.184). Post hoc analysis found that the peak benefit was significantly lower at posttest than at pretest for the TG [21.47% vs. 9.18%, *t*_(14)_ = −1.879, *p* = 0.012, *d* = −0.485], whereas it remained comparable for the CG [19.46% vs. 20.96%, *t*_(15)_ = 0.719, *p* = 0.573, *d* = 0.180]. These results suggest that the pattern of audiovisual integration is altered by training.

###### 3.1.2.3.2 Attention

There was a significant main effect of cue [*F*(2, 58) = 59.134, *p* < 0.001, *η_p_^2^* = 0.671], with faster responses for spatial cue trials compared to no cue and center cue trials (spatial > center > no, all *ps* ≤ 0.042). Additionally, there was a main effect of target [*F*(2, 58) = 216.582, *p* < 0.001, *η_p_^2^* = 0.882], with faster responses to congruent targets than incongruent targets, and a main effect of test [*F*(1, 29) = 5.797, *p* = 0.023, *η_p_^2^* = 0.167], indicating faster responses at posttest than at pretest. The attention networks (alerting, orienting, and executive control) were calculated (Figure 3E), but no significant differences were found between pretest and posttest (*all ps* ≥ 0.051).

### 3.2 EEG results

#### 3.2.1 Training effect

**Figure 4:**
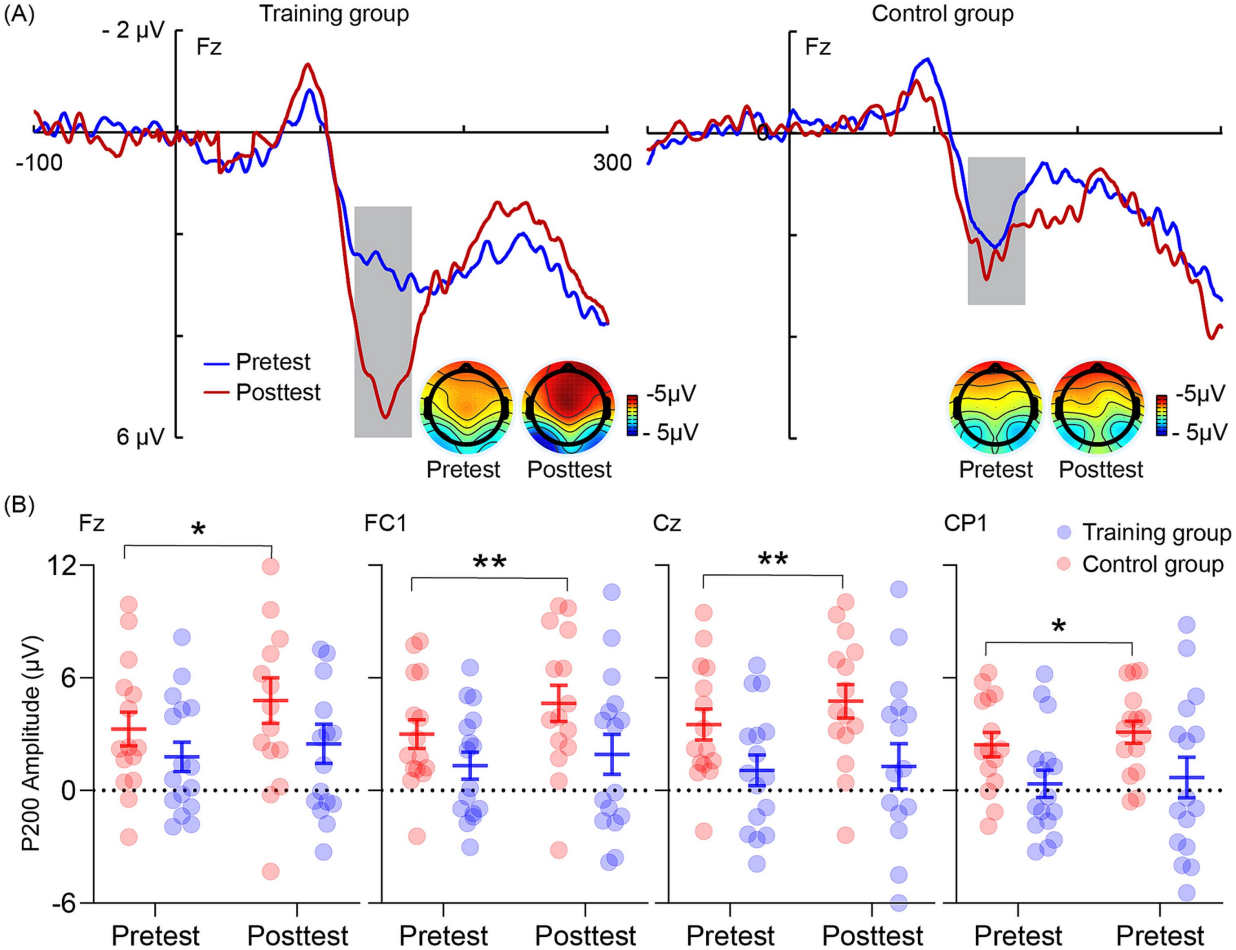
Grand-averaged event-related potentials for the pre and posttests are shown for the training and control groups **(A)**. The P200 amplitude (120–160 ms) was significantly greater at posttest for the training group at Fz, FC1, Cz, and CP1 **(B)**. Error bars represent the standard error of the mean (SEM) across participants. ^*^p < 0.05, ^**^p < 0.01.

Significant main effects of test and ROI were observed (Table 3). In addition, a significant test × group interaction was also found. Post hoc analysis revealed that the TG showed higher P200 amplitudes at the Fz [*t*_(14)_ = 2.899, *p* = 0.012, *d* = 0.749], FC1 [*t*_(14)_ = 3.741, *p* = 0.002, *d* = 0.966], Cz [*t*_(14)_ = 3.657, *p* = 0.003, *d* = 0.944] and CP1 [*t*_(14)_ = 2.269, *p* = 0.040, *d* = 0.586] at posttest compared to the pretest (Figure 4; Supplementary Figure 1). In contrast, the CG showed no significant changes across any electrode (all *ps* ≥ 0.157). No significant differences were observed between the TG and CG at any electrode at pretest (all *ps* ≥ 0.120); however, at posttest, the TG exhibited significantly higher P200 amplitudes than the CG at Fz [*t*_(29)_ = 2.109, *p* = 0.044, *d* = 0.758], FC1 [*t*_(29)_ = 2.116, *p* = 0.043, *d* = 0.760], and Cz [*t*_(29)_ = 2.284, *p* = 0.030, *d* = 0.821], with a trend toward significance at CP1 [*t*_(29)_ = 1.922, *p* = 0.064, *d* = 0.691].

**Table 3:** Mixed-design ANOVA results for P200 amplitude during the visuospatial 1-back task (120–160 ms).

Main Effects and Interactions	*df*	F	*p*	*η_p_^2^*
Group	1, 29	3.784	0.061	0.115
Test	1, 29	9.176	**0.005**	0.240
Test × Group	1, 29	12.410	**0.030**	0.140
ROI	3, 87	9.377	**<0.001**	0.244
ROI × Group	3, 87	1.241	0.292	0.041
Test × ROI	3, 87	2.103	0.147	0.068
Test × ROI × Group	3, 87	0.644	0.480	0.022

Values shown in bold denote statistically significant differences (*p* < 0.05).

#### 3.2.2 Far transfer

##### 3.2.2.1 Audiovisual integration

**Figure 5:**
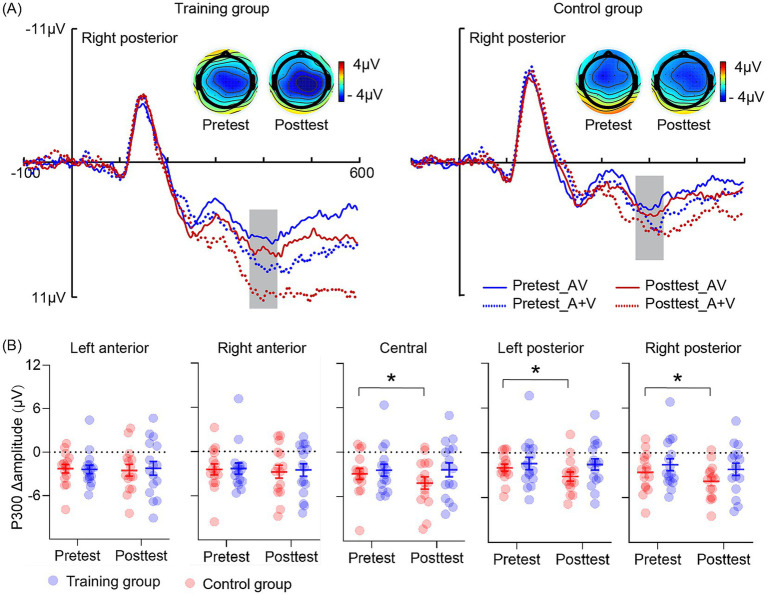
**(A)** Grand-average event-related potentials (ERPs) from the central region (mean amplitudes across electrodes P4, CP6, and CP2), along with a topographic map illustrating audiovisual integration, computed as the difference waveform [ERP_audiovisual_ − ERP_(auditory + visual)_]. **(B)** P300 difference wave amplitudes (*Δ* amplitude, 370–430 ms) reflecting audiovisual integration, with more negative values indicating reduced ERP amplitudes in the audiovisual condition relative to the auditory-only and visual-only conditions. Error bars represent the standard error of the mean (SEM) across participants. ^*^p < 0.05.

Significant main effects of test and ROI were observed (Table 4). In addition, a significant test × group interaction was also found. Post hoc analysis showed that, within the TG, *Δ* amplitudes significantly increased at the central [*t*_(14)_ = −2.161, *p* = 0.026, *d* = −0.558] and right posterior [*t*_(14)_ = −2.368, *p* = 0.019, *d* = −0.611], ROIs at posttest relative to the pretest, with a trend toward significance at the left posterior ROI [*t*_(14)_ = −1.754, *p* = 0.058, *d* = −0.453] (Figure 5; Supplementary Figure 2). No significant changes were found at the left anterior [*t*_(14)_ = −0.728, *p* = 0.739, *d* = −0.188] and left posterior [*t*_(14)_ = −0.475, *p* = 0.642, *d* = −0.123] ROIs. In contrast, the CG showed no significant differences across any ROIs (all *ps* ≥ 0.618). Further comparison between groups revealed no significant differences across ROIs at pretest (all *ps* ≥ 0.243). However, at posttest, the TG showed significantly greater Δ amplitudes than the CG at the central [*t*_(29)_ = −2.060, *p* = 0.029, *d* = −0.740], left posterior [*t*_(29)_ = −1.820, *p* = 0.046, *d* = −0.654], and right posterior [*t*_(29)_ = −2.191, *p* = 0.024, *d* = −0.787] ROIs, while no significant differences were observed at the left anterior [*t*_(29)_ = −0.449, *p* = 0.626, *d* = −0.161] and right anterior [*t*_(29)_ = −0.527, *p* = 0.536, *d* = −0.189] ROIs.

**Table 4:** Mixed-design ANOVA results for P300 Δ amplitude during audiovisual integration (370–430 ms).

Main Effects and Interactions	*df*	F	*p*	*η_p_^2^*
Group	1, 29	2.075	0.204	0.071
Test	1, 29	11.053	**0.012**	0.137
Test × Group	1, 29	4.373	**0.034**	0.098
ROI	4, 116	13.825	**0.006**	0.117
ROI × Group	4, 116	0.949	0.421	0.032
Test × ROI	4, 116	2.228	0.107	0.071
Test × ROI × Group	4, 116	0.296	0.829	0.010

Values shown in bold denote statistically significant differences (*p* < 0.05).

##### 3.2.2.2 Attention

###### 3.2.2.2.1 Target N100 alerting and orienting effects

There was a significant main effect of cue [*F*(2, 58) = 26.131, *p* = 0.028, *η_p_^2^* = 0.183], with greater N100 amplitudes observed in the spatial cue condition compared to the center and no cue conditions (spatial > center > no cue, all *ps* ≤ 0.013) (Figure 2A). A follow-up 2 (group) × 2 (test) mixed-design ANOVA for alerting and orienting effects revealed no significant main effects or interactions (all *ps* ≥ 0.712). Taken together with the behavioral results, these findings suggest that WM training improved response speed, but did not modulate the alerting or orienting attention networks.

###### 3.2.2.2.2 Target P300 conflict effect

There was a significant interaction between test and group [*F*(1, 29) = 3.550, *p* = 0.048, *η_p_^2^* = 0.109]. Post hoc analysis indicated a significantly greater conflict effect at posttest compared to the pretest for the TG [*t*_(14)_ = −1.957, *p* = 0.011, *d* = −0.524] but not for the CG [*t*_(15)_ = −0.877, *p* = 0.557, *d* = −0.231] (Figure 2B). Additionally, the conflict effect amplitude was significantly greater for the TG than CG at posttest [*t*_(29)_ = 1.615, *p* = 0.023, *d* = 0.592], while no significant group difference was observed at pretest [*t*_(29)_ = 0.518, *p* = 0.440, *d* = 0.192]. These results suggest enhanced executive control and faster conflict processing following WM training.

## 4 Discussion

The primary objective of this study was to examine training-related changes following adaptive visuospatial n-back training in audiovisual integration and attention networks. Partly consistent with our hypothesis, task difficulty increased across training sessions and the trained 1-back elicited greater P200 amplitudes in the fronto-central, central, and centro-parietal regions, suggesting learning and neural modulation within the trained task. For near transfer, the TG performed better on the untrained Corsi block test, suggesting a potential benefit for visuospatial WM. However, evidence for transfer was not consistent across outcomes. For audiovisual integration, the race model peak benefit decreased after training, whereas P300-related audiovisual activity increased, indicating a dissociation between behavioral facilitation and late-stage neural processing. A similar dissociation was observed in the attention network task, where no reliable behavioral improvement was found despite increased parietal P300 amplitudes during incongruent stimulus processing.

In line with previous studies (Bherer et al., 2008; Borella et al., 2014; Borella et al., 2010; Dahlin et al., 2008b; Guo et al., 2023; Heinzel et al., 2016; Heinzel et al., 2014; Iordan et al., 2020; Iordan et al., 2021; Jaeggi et al., 2008; Li et al., 2021; Pergher et al., 2018; Spironelli and Borella, 2021; von Bastian et al., 2013), the present findings suggest that training was accompanied by a progressive increase in task difficulty level. This gradual adjustment is a defining feature of adaptive training paradigms, intended to maintain optimal challenge by matching cognitive demands to participants’ evolving performance levels (Fraulini et al., 2024; Pelosi et al., 2024). In the current study, EEG data from the 1-back task revealed that the TG exhibited greater P200 amplitudes in the fronto-central, central, and centro-parietal regions. This pattern may indicate enhanced perceptual discrimination and/or improved attentional modulation associated with the trained task. As match trials require participants to determine whether the current stimulus corresponds to a previously encoded one, an increased P200 amplitude could suggest enhanced early differentiation between matching and nonmatching stimuli (Cowan, 2022). Previous studies have also associated the P200 component with early attentional modulation of perceptual processing (Key et al., 2005), indicating that the observed enhancement may reflect more effective allocation of attentional resources. Alternatively, the enhanced P200 amplitude might reflect more efficient sensory processing or increased task engagement, both of which could contribute to task performance. These interpretations align with prior research demonstrating increased frontal oscillatory activity in older adults following cognitive training (Spironelli and Borella, 2021), and enhanced neural responses during spatial 3-back tasks in individuals with multiple sclerosis (Turtola and Covey, 2021). Taken together, the present findings suggest that adaptive visuospatial n-back training may modulate perceptual and attentional processes during low-load WM tasks, potentially accounting for the increased task difficulty achieved across training sessions. However, no significant improvements in behavioral accuracy were detected. One possible explanation is the relatively short training duration, which may have been insufficient to elicit measurable behavioral changes in this older adult population. Thus, the observed neurophysiological changes may reflect early-stage neural adaptation in the absence of detectable behavioral gains.

The better performance observed in the Corsi block test may reflect a potential near transfer effect on visuospatial WM. This enhancement could be attributed to shared cognitive processes between the two tasks, particularly those involving visuospatial WM, as supported by evidence from EEG and fMRI studies (Iordan et al., 2020; Miró-Padilla et al., 2020; Salmi et al., 2018). These findings suggest that n-back training may strengthen visuospatial WM processes, thereby partially accounting for the performance improvements observed in the Corsi block test (Heinzel et al., 2016). Additionally, the adaptive nature of the training, characterized by the continuous adjustment of task difficulty level based on individual performance, may have helped maintain an optimal level of cognitive challenge throughout the intervention. However, given the ongoing debate on cognitive training transfer, the increased Corsi block span should be interpreted cautiously as a potential near transfer effect, because the Corsi block test and the training task share visuospatial WM demands (Fraulini et al., 2024; Pelosi et al., 2024).

In line with prior EEG studies (Wang and Covey, 2020; Wei et al., 2022), the present findings suggest that visuospatial WM training may be associated with changes in neural processing during conflict trials in attention network test, as reflected by increased P300 amplitudes in the parietal region at posttest in the training group. The P300 component is commonly associated with high-level cognitive functions such as attention allocation, stimulus evaluation, and the updating of WM representations (Linden, 2005). Executive control refers to the regulation of cognitive functions such as WM, cognitive flexibility, and inhibitory control (Fan et al., 2002). Higher P300 amplitudes in the parietal region during conflict stimulus processing may indicate training-related changes in the neural processing of executive control (Awh et al., 2006; Borella et al., 2014; Pergher et al., 2018). This pattern may reflect altered allocation of cognitive resources during the resolution of competing task demands and response selection (Fan et al., 2002). Additionally, the increased P300 amplitude may reflect changes in conflict detection and resolution, sustained attention, or task engagement. This pattern is broadly consistent with previous evidence suggesting that n-back training may modulate neural processing related to conflict monitoring (Wang and Covey, 2020). Given the role of the parietal cortex in integrating sensory input and supporting complex cognitive operations, enhanced P300 activity in this region may reflect training related modulation of neural processing during conflict trials (Neuhaus et al., 2010).

However, the absence of significant behavioral improvements in attention network performance suggests that the ERP changes did not translate into measurable behavioral gains. Therefore, the increased parietal P300 amplitude should be interpreted as a neural change associated with conflict processing rather than as direct evidence of improved attention network performance. This discrepancy may be related to the relatively short duration or limited intensity of the training protocol, and more extended interventions may be required to elicit observable behavioral effects (Borella et al., 2025; Jaeggi et al., 2008; Liu et al., 2024). Notably, no significant changes after training were observed in either the behavioral or electrophysiological indices of the alerting and orienting components. This may reflect the specificity of training effects, as executive control is primarily associated with the dopaminergic system, whereas alerting and orienting are more closely linked to the noradrenergic and cholinergic systems, respectively (Petersen and Posner, 2012). As such, training designed to engage WM and executive control may not necessarily influence neural circuits underlying other attentional subsystems. Alternatively, the absence of significant changes may also be due to methodological limitations, such as the relatively small sample size and the brief training duration, both of which may have reduced the sensitivity to detect subtle effects in alerting and orienting networks.

Furthermore, to our knowledge, this is the first study to report a potential transfer effect of WM training on audiovisual integration in older adults. The training group showed reduced behavioral benefits from audiovisual integration, as indexed by the race model, but increased P300-related audiovisual integration effects, as reflected by the amplitude difference between the audiovisual condition and the summed auditory and visual conditions [AV − (A + V)]. The behavioral index derived from the race model captures the extent to which audiovisual responses exceed the facilitation predicted by independent unimodal processing (Stein, 2012). Under the conventional interpretation of the race model, a reduced peak benefit indicates smaller behavioral facilitation from audiovisual stimulation beyond unimodal processing and may therefore suggest weaker behavioral multisensory integration. Thus, the decreased race model violation observed after training should not be interpreted as behavioral evidence for enhanced audiovisual integration. However, an alternative and more tentative interpretation is that the reduced behavioral benefit may reflect reduced reliance on audiovisual redundancy after training, particularly if unimodal processing became more efficient (Tye-Murray et al., 2010). One possible explanation is related to the principle of inverse effectiveness. According to this principle, multisensory benefits are typically greater when unimodal processing is weak or inefficient, whereas the additional behavioral benefit from audiovisual stimulation may diminish as unimodal processing becomes more efficient (Stein, 2012; Tye-Murray et al., 2010). In the present study, adaptive visuospatial n-back training may have modulated visuospatial WM updating, sustained attention, and the allocation of task-relevant cognitive resources (Pappa et al., 2020; Pergher et al., 2018). These changes may have supported unimodal stimulus processing, thereby reducing the additional behavioral advantage provided by audiovisual redundancy.

In contrast to the behavioral index, the increased P300 audiovisual effect may reflect greater late-stage neural engagement during audiovisual stimulus evaluation rather than enhanced behavioral integration. This neural change may appear inconsistent with the reduced behavioral peak benefit, but the two measures may capture different levels of audiovisual processing. The race model primarily reflects overt behavioral facilitation beyond unimodal processing, whereas P300 activity is more closely related to stimulus evaluation, attentional resource allocation, and the updating of task-relevant information (Linden, 2005). These cognitive operations overlap closely with the processes engaged by adaptive visuospatial n-back training, including WM updating, sustained attention, and attentional control (Baddeley, 2012; Oberauer, 2019). This interpretation is also consistent with fMRI evidence showing that WM training can upregulate activity in the dorsolateral and ventrolateral prefrontal cortices, regions involved in cognitive control and the top-down modulation of multisensory processing (Salmi et al., 2018). Similar cross-domain transfer effects have also been reported in younger adults following dual n-back training, providing indirect support for the possibility that WM training may influence untrained cognitive and perceptual processes (Guo et al., 2023; Li et al., 2021). Thus, the reduced behavioral benefit and increased P300 audiovisual effect are not necessarily contradictory. Instead, they may indicate that adaptive visuospatial n-back training was associated with reduced behavioral dependence on audiovisual redundancy while showing greater late-stage neural engagement during audiovisual information processing. Nevertheless, given the debate regarding the robustness and generalizability of far transfer effects in n-back training, this interpretation should be considered tentative. Future studies with larger samples, active control conditions, and longer follow-up assessments are needed to further clarify how visuospatial WM training affects behavioral and neural indices of audiovisual integration.

Overall, the present findings suggest that adaptive visuospatial n-back training in older adults was associated with improved performance on the trained task and with selective changes in untrained outcomes, including Corsi block span, P300 indices of audiovisual integration, and neural processing during executive control. Rather than providing definitive evidence of broad cognitive transfer, these findings should be interpreted as preliminary evidence for changes in domains that share partially overlapping processes with the training task. According to the STAC-r, such changes may reflect the engagement of compensatory scaffolding mechanisms through structured cognitive engagement, which could help older adults cope with age-related neural and cognitive challenges. Nevertheless, given the debate regarding the robustness and generalizability of transfer effects in cognitive training, further studies with larger samples, active control groups, longer training protocols, and follow-up assessments are needed to determine the durability, specificity, and practical significance of these effects. Taken together, this study suggests that adaptive visuospatial n-back training may be associated with behavioral and electrophysiological changes relevant to cognitive functioning in later life, although its broader transfer potential remains to be established.

## 5 Limitations

First, the relatively small sample size (*N* = 31) may limit the generalizability and statistical power of the findings. The modest sample size also restricted the analysis of individual differences, as potentially moderating variables, such as baseline cognitive ability, education level, and other demographic factors, were not examined. Second, the training duration was relatively brief, with the intervention lasting only one week. Although the present findings suggest short-term training-related changes, future studies with longer intervention periods and follow-up assessments are needed to determine whether these effects are stable and sustained over time. Third, the use of a non-contact control group may not fully control for placebo effects, expectancy effects, or nonspecific effects related to researcher contact and task engagement. Therefore, future studies should include an active control condition matched for training duration, experimenter contact, and task demands to strengthen the causal interpretation of training-specific effects.

## Data Availability

The datasets and materials generated during the current study are available from the corresponding author upon reasonable request.
