# Supplementary material for: Adaptive visuospatial n-back training improves audiovisual integration and executive control in older adults
Source: Front Psychol. 2026 Jun 19;17:1801385. doi: 10.3389/fpsyg.2026.1801385 (PMC13328510; doi:10.3389/fpsyg.2026.1801385)
Supplement: Supplementary file 1 [file Supplementary_file_1.DOCX]

**Supplementary Materials**


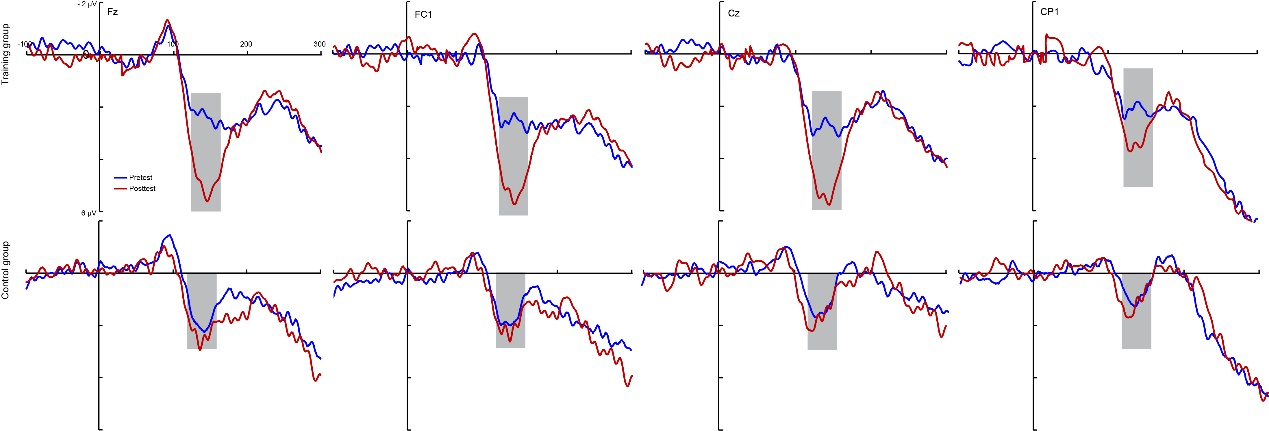


**Supplementary Figure 1**: Grand-averaged event-related potentials for the P200 component (120–160 ms), highlighted with gray squares, in the pre and posttest conditions for both the training and control groups.


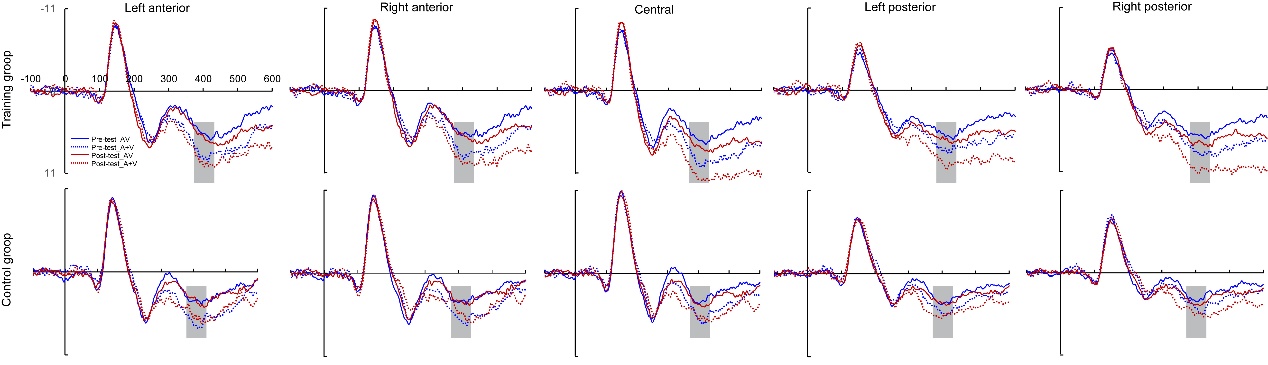


**Supplementary Figure 2**: Grand-averaged event-related potentials (ERPs) for the P300 component (370–430 ms), highlighted with gray squares, in the pre and posttest conditions for both the training and control groups. The grand-averaged ERPs for the left anterior region represent the mean amplitudes of FC5, F3, and FC1; the right anterior region includes the mean amplitudes of FC6, F4, and FC2; the central region comprises the mean amplitudes of C3, Cz, and C4; the left posterior region corresponds to the mean amplitudes of CP5, P3, and CP1; and the right posterior region represents the mean amplitudes of CP6, P4, and CP2.
